# Deferoxamine prevents dexamethasone-induced muscle atrophy by reducing MuRF1 and atrogin-1

**DOI:** 10.3389/fphar.2025.1582216

**Published:** 2025-07-09

**Authors:** Jae-Yeop Jeong, Youngho Son, Youngha Kim, Nami Lee, Yujin Kim, Yu Jung Heo, Sung-E. Choi, Jaemyung Choi, Seung Jin Han, Jayoung Jeon, Hae Jin Kim, Kwan-Woo Lee

**Affiliations:** ^1^ Department of Endocrinology and Metabolism, Ajou University School of Medicine, Suwon, Republic of Korea; ^2^ Institute of Medical Science, Ajou University School of Medicine, Suwon, Republic of Korea; ^3^ Department of Physiology, Ajou University School of Medicine, Suwon, Republic of Korea

**Keywords:** muscle atrophy, dexamethasone, deferoxamine, MuRF1, atrogin-1

## Abstract

**Introduction:**

Muscle atrophy, commonly triggered by glucocorticoids such as dexamethasone (DEX), involves increased protein degradation via the ubiquitin–proteasome system. Recent findings suggest that iron imbalance can also induce muscle atrophy. However, there have been no reports indicating that DEX causes intracellular iron imbalance leading to muscle atrophy. This study evaluated whether DEX causes iron imbalance-mediated muscle atrophy and whether deferoxamine (DFO), an iron chelator, can protect against DEX-induced muscle atrophy, exploring the underlying mechanisms *in vitro* and *in vivo*.

**Method:**

Differentiated C2C12 myotubes were exposed to DEX, with or without DFO, to evaluate morphological changes, expression of muscle-specific ubiquitin ligases (atrogin-1 and MuRF1), and related signaling pathways via quantitative reverse transcription polymerase chain reaction, Western blotting, and immunocytochemistry. Intracellular iron accumulation was quantified using fluorescence imaging. Additionally, C57BL/6J mice were administered intraperitoneal injections of DEX, with or without DFO, every other day for 12 days. Muscle function was assessed by grip strength, and muscle mass and fiber size were measured histologically.

**Results:**

DEX significantly induced muscle atrophy in C2C12 myotubes, elevating intracellular iron and upregulating atrogin-1 and MuRF1 via increased nuclear translocation of FOXO3a and expression of KLF15. DFO treatment prevented these effects by restoring the iron balance, enhancing AKT phosphorylation, inhibiting FOXO3a nuclear translocation, and reducing KLF15 expression. Consistently, animal experiments demonstrated that DFO administration effectively preserved grip strength, tibialis anterior muscle mass, and muscle fiber size in DEX-treated mice. Furthermore, DFO treatment restored insulin-like growth factor 1 and myostatin expression levels altered by DEX.

**Discussion:**

DFO effectively ameliorates DEX-induced muscle atrophy by modulating the AKT/FOXO3a and KLF15 signaling pathways and restoring the intracellular iron balance. These findings highlight DFO as a potential therapeutic agent for glucocorticoid-induced muscle atrophy.

## 1 Introduction

Skeletal muscles are the largest organ system in the body, making up approximately 45%–55% of body mass, and they play an important role in regulating the basal metabolic rate (Periasamy et al., 2017). Under normal conditions, muscle protein synthesis and breakdown rates are in balance, but when atrophy occurs, the rate of protein degradation exceeds the rate of protein synthesis (Breen and Phillips, 2011). Muscle atrophy is caused by various physiological and pathological conditions such as inactivity, cancer, aging, and diabetes (Jun et al., 2023). Such skeletal muscle atrophy has become a global social problem because of the associated decrease in quality of life. Many studies focusing on the treatment of muscle atrophy are being conducted, but there is still no effective drug treatment and no clearly established therapeutic mechanism.

The pathogenesis of muscle atrophy is very complex but can be explained by several pathophysiological processes, including oxidative stress and inflammation, which activate signaling pathways such as the ubiquitin–proteasome system (UPS), the autophagy–lysosome system, and mTOR (Sandri, 2013). Recently, proteolysis via the UPS has been reported as an important mechanism affecting muscle atrophy (Pang et al., 2023). The UPS is involved in muscle protein turnover triggered during most catabolic processes that induce muscle atrophy (Bonaldo and Sandri, 2013). In the process of ubiquitination, ATP-dependent muscle-specific E3 ligases such as muscle ring finger 1 (MuRF1) and atrogin-1 (also known as MAFbx) play a central role (Bodine et al., 2001; Gomes et al., 2001).

Glucocorticoids (GCs) are endogenous hormones secreted under various stress conditions, such as starvation, denervation, and diabetes, and they are widely used in the study of catabolic muscle atrophy (Schakman et al., 2013). In skeletal muscle, GC excess has been reported to inhibit glucose uptake and utilization by antagonizing the insulin response, ultimately leading to mitochondrial dysfunction and muscle atrophy (Kuo et al., 2013). These effects of GCs are thought to be mediated by GC receptors (GRs). Synthetic GCs used for treatment include hydrocortisone, prednisone, and dexamethasone (DEX) (Trikudanathan and McMahon, 2008). DEX has been used to induce muscle atrophy through proteolysis (Sandri et al., 2004). High concentrations of DEX can upregulate myostatin expression and increase the expression of the E3 ubiquitin ligase, atrogin-1, and MuRF1. DEX regulates the expression of Krüppel-like factor 15 (KLF15), a member of the zinc finger transcription factor family of proteins. KLF15 acts as a catabolic modulator of skeletal muscle via the direct transcriptional upregulation of MAFbx1/atrogin-1 and MuRF1, which are associated with muscle atrophy. FOXO3a is a member of the forkhead family of transcription factors and is primarily regulated by the PI3K/Akt (PKB) signaling pathway. Wang et al. (2018) reported that *Morinda officinalis* improves DEX-induced muscle atrophy, apparently by regulating the AKT/mTOR/FOXO3a signaling pathway.

Prolonged DEX treatment disrupts the iron metabolism balance in several tissues (Li et al., 2017). Iron is an important element involved in many physiological and pathologic processes, including redox balance, inflammation, and metabolism (Roemhild et al., 2021). Studies of the relationship between muscle iron metabolism and muscle physiology have reported that iron deficiency impairs skeletal muscle functioning in the context of mitochondrial function during energy metabolism (Dziegala et al., 2018; Leermarkers et al., 2020). Conversely, excess iron accumulation in muscles triggers iron-dependent oxidative stress, producing reactive oxygen species (Ikeda et al., 2016; Halon-Golabek et al., 2019), suggesting another route by which iron imbalance can lead to skeletal muscle atrophy. However, the full process and exact molecular mechanisms of iron imbalance-induced muscle atrophy remain to be elucidated. In a previous study, we found that palmitate and iron donors simultaneously increased intracellular iron and induced insulin resistance by activating Jun N-terminal kinase (JNK) (Cui et al., 2019). Deferoxamine (DFO) is an iron chelator—a hexadentate siderophore molecule that has high affinity for iron (Richardson, 2002). DFO dramatically inhibited palmitate-induced insulin resistance. In this study, we investigated the molecular mechanisms of the DEX-induced muscle atrophy in C2C12 myotubes and protective effects of DFO in a mouse sarcopenia model.

## 2 Materials and methods

### 2.1 Reagents

Dexamethasone (DEX; 1756), Deferoxamine (DFO; 9533), dimethyl sulfoxide (DMSO; 2438) were purchased from Millipore Sigma (Burlington, MA, USA). Chemicals were dissolved in appropriate media or dimethyl sulfoxide and then used at the recommendation concentrations. Anti-phospho-eIF2 α (Ser51) (9721), anti-total-eIF2 α (9722), anti-ATF4 (11815), anti-phospho-SAPK/JNK (Thr183/Tyr185) (9251), anti-phospho-CaMKK2 (Ser495) (16737), anti-pro-caspase3 (9662), anti-cleaved caspase3 (9661), anti-p62 (5114), anti-LC3β (2775), anti-phospho-FoxO3a (Ser253) (13129), anti-total-FoxO3a (12829), anti-phospho-Akt (Ser473) (9271), anti-total-Akt (9272), anti-phospho-p70 S6K (9208), anti-phospho-4E-BP1 (Ser65) (9451), anti-total-4E-BP1 (9452) antibodies and FerroOrange (36104S) were obtained from Cell Signaling Technology (Danvers, MA, USA). Anti-GDF8 (sc134345), anti-KLF15 (sc271675), anti-MuRF1 (sc398608), anti-Ubiquitin (sc8017) antibodies were obtained from Santa Cruz Biotechnology (Dallas, TX, USA). Anti-Atrogin1 (ab168372) antibodies were obtained from Abcam (Cambridge, United Kingdom). Anti-total-p70 S6K (ADI-KAP-CC035) antibody was purchased from Enzo Life Science (Farmingdale, NY, USA). Anti-β-actin (A300-491A) antibody was purchased from Bethyl Laboratories (Montgomery, TX, USA).

### 2.2 Cell culture

Undifferentiated mouse skeletal muscle myoblasts (C2C12) were obtained from American Type Culture Collection (ATCC, Manassas, VA) and were maintained in high-glucose (1 g/L) Dulbecco’s modified Engle’s medium (DMEM) supplemented with 10% fetal bovine serum and antibiotics (100 IU/mL penicillin and 10 μg/mL streptomycin) at 37°C in a humidified atmosphere of 5% CO_2_. C2C12 myoblasts were differentiated for 5 days in high-glucose (1 g/L) Dulbecco’s modified Engle’s medium (DMEM) supplemented with 2% horse serum and antibiotics (100 IU/mL penicillin and 10 μg/mL streptomycin) at 37°C in a humidified atmosphere of 5% CO_2_.

### 2.3 Immunoblotting

C2C12 myotubes were suspended in RIPA buffer [150 mM NaCl, 1% NP-40, 0.5% deoxycholate, 0.1% sodium dodecyl sulfate (SDS), 50 mM Tris–Cl, pH 7.5, and protease inhibitor cocktail (Roche Applied Science, Mannheim Germany)] and incubated on ice for 30 min. Whole proteins were extracted by differential centrifugation (10,000*g*, 10 min), and protein concentrations in lysates were determined using a protein assay kit (Bio-Rad, Hercules, CA, USA). An equal volume of 2 × SDS sample buffer (125 mM Tris–Cl, pH 6.8, 4% SDS, 4% 2-mercaptoethanol, 20% glycerol) was added to the cell lysates, and equivalent amounts of protein (5 μg) were loaded onto 10%–15% polyacrylamide gels, electrophoresed, and electrophoretically transferred onto polyvinylidene fluoride membranes (Millipore, Bedford, MA, USA). After blocking the membranes with 5% skimmed milk for 30 min, target antigens were reacted with primary antibodies, followed by secondary antibodies (horseradish peroxidase-conjugated anti-mouse IgG or anti-rabbit IgG). Immunoreactive bands were visualized by enhanced chemiluminescence (Amersham Pharmacia Biotech, Arlington Height, IL, USA).

### 2.4 Reverse transcriptase-polymerase chain reaction (RT-PCR)

Total RNA from cells and tissues was extracted with RNAiso Plus reagent (TaKaRa Bio, Shiga, Japan). cDNA was synthesized using the TaKaRa PrimeScript™ RT Master Mix (TaKaRa Bio). The primer sets for PCR amplification are listed in Supplementary Table 1. Quantitative real-time PCR was performed with TB Green^®^ Premix Ex Taq™ (TaKaRa Bio) using a QuantStudio 1 (Thermo Fisher Scientific, MA, United States) instrument. Relative quantities of amplified DNA were analyzed using the software bundled with the QuantStudio 1 instrument and normalized to mouse 36B4 mRNA levels.

### 2.5 Phalloidin and DAPI staining

C2C12 myotubes were fixed with 3.7% formaldehyde solution for 10min. Subsequently, samples were incubated in PBS with 0.1% Triton X-100, 2.5% Alexa fluor 488-conjugated phalloidin (Thermo fisher Scientific), and 2.5% DAPI (Thermo Fisher Scientific) at room temperature in the dark for 10–30 min. Phalloidin stains filamentous actin, DAPI labels cell nuclei. After washing samples three times with PBS, they were mounted on glass slides. Fluorescence was observed under a confocal microscope (Nikon, Tokyo, Japan).

### 2.6 Immunocytochemistry

C2C12 myotubes were fixed with 3.7% formaldehyde solution for 10 min. Subsequently, samples were incubated in PBS with 0.1% Triton X-100 at room temperature for 5 min, 0.2% FoxO3a primary antibodies (12829,Cell signaling) at −40°C for overnight, Alexa Fluor 488 goat anti-rabbit IgG (H + L) (A11034, invitrogen) at −40°C for 2 h and 0.2% DAPI (Thermo Fisher Scientific) at room temperature in the dark for 10 min FoxO3a primary and secondary antibodies stains FoxO3a proteins, DAPI labels cell nuclei. After washing samples three times with PBS, they were mounted on glass slides. Fluorescence was observed under a confocal microscope (Nikon, Tokyo, Japan).

### 2.7 Detection of intracellular Fe^2+^

To detect intracellular Fe^2+^, C2C12 myotubes were stained with FerroOrrange. Briefly, C2C12 myoblast cells were seeded in a 12-well plate and induced to differentiate for 5 days. After differentiation, C2C12 myotubes were treated with 20 µM dexamethasone for 24 h. Subsequently, the cells were washed with PBS and incubated with FerroOrange at a final concentration of 1 μmol/L for 30 min at 37°C. Following the incubation, the cells were washed three times with PBS, and intracellular Fe^2+^ levels were visualized using an EVOS M5000 imaging system (Thermo Fisher Scientific inc., Waltham, MA, United states) and were quantified using SpectraMax iD3 (Molecular Devices, San Jose, CA, United states).

### 2.8 Animal study

Six-week-old male C57BL/6J mice were obtained from GemPharmatech. Co. (Nanjing, China). These mice were housed in a temperature-controlled room at (22°C ± 2°C) with a 12/12-h light/dark cycle and fed *ad libitum.* Following 2 weeks of adaptation, mice were randomly assigned into the following three groups: 1) DMSO-injected control group (CON); 2) Dexamethasone-injected group (DEX); 3) Dexamethasone and deferoxamine-treated group (DEX/DFO). Treated groups were injected intraperitoneally every other day for 12 days with DMSO or dexamethasone and deferoxamine. All animal care and treatments were conducted according to the Ajou Institutional Animal Cere guidelines and were approved by Ajou Institutional Animal Care Committee (Permission Number: 2023-0092).

### 2.9 Grip strength test

Grip strength was assessed using a grip strength meter with grid (Bioseb, France) to measure the forelimb strength of each group. Each mouse was allowed to grasp the grid with its forelimbs while being gently pulled backwards by the tail until the grip was released. The test was performed three times for each mouse, with a 30-s rest between trials, and the average value was used as the grip strength score. The results were normalized to body weight and analyzed statistically.

### 2.10 Hematoxylin and Eosin staining

TA muscle tissues fixed with PBS containing 4% paraformaldehyde were embedded in paraffin, sliced into 5-μm thick sections, mounted onto slides, and sequentially stained in hematoxylin and eosin solution (Abcam, Cambridge, UK) according to standard procedures.

### 2.11 Statistical analysis

All experiments were repeated at least three times. All data are expressed as the mean ± SE and were analyzed using GraphPad Prism 9.2.0 (GraphPad Software Inc., San Diego, CA, United States). One-way analysis of variance (ANOVA) with the Bonferroni *post hoc* test was used. *p* values < 0.05 were considered statistically significant.

## 3 Results

### 3.1 DEX increases intracellular iron levels in C2C12 myotubes

To mimic GC-induced muscle atrophy, differentiated C2C12 myotubes were treated with 20 µM DEX as a GC activator for 24 h. The myotubes were then fixed and stained with phalloidin and DAPI (Supplementary Figure 1A). The DEX-treated C2C12 myotubes were smaller, indicating that DEX had induced muscle atrophy (Supplementary Figure 1B).

**FIGURE 1:**
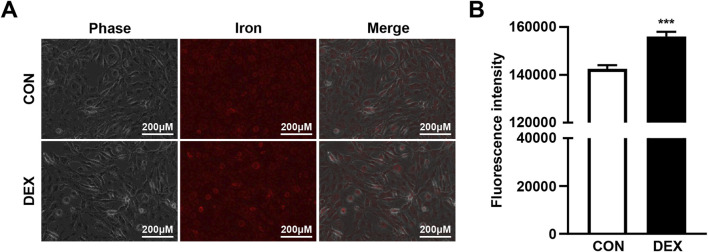
DEX treatment increases intracellular iron levels in C2C12 myotubes. **(A)** C2C12 myoblasts were differentiated for 5 days and treated with 20 µM DEX for 24 h. After treatment, intracellular iron levels were visualized using a fluorescent iron-staining dye and fluorescence microscopy. **(B)** Fluorescence intensity was quantified by spectrophotometry. Data are expressed as mean ± SEM. Statistical significance in **(B)** was determined using a t-test. ***p < 0.001 vs. control (CON).

As previous research has demonstrated that palmitic acid-induced muscle atrophy and insulin resistance are linked to intracellular iron overload, which exacerbates metabolic disturbances in skeletal muscle cells (Cui et al., 2019), we investigated whether DEX treatment alters intracellular iron levels in C2C12 myotubes. Fluorescence microscopy using an iron ion-specific staining dye revealed that intracellular iron levels increased significantly following the DEX treatment compared to untreated controls (Figure 1A). This observation was confirmed by quantitative analysis of fluorescence intensity, which demonstrated a marked increase in intracellular iron accumulation in DEX-treated cells (Figure 1B). These findings suggest that DEX not only induces morphological changes indicative of muscle atrophy but also disrupts intracellular iron homeostasis.

### 3.2 DEX induces C2C12 myotube atrophy by inducing atrogin-1 and MuRF1

**FIGURE 2:**
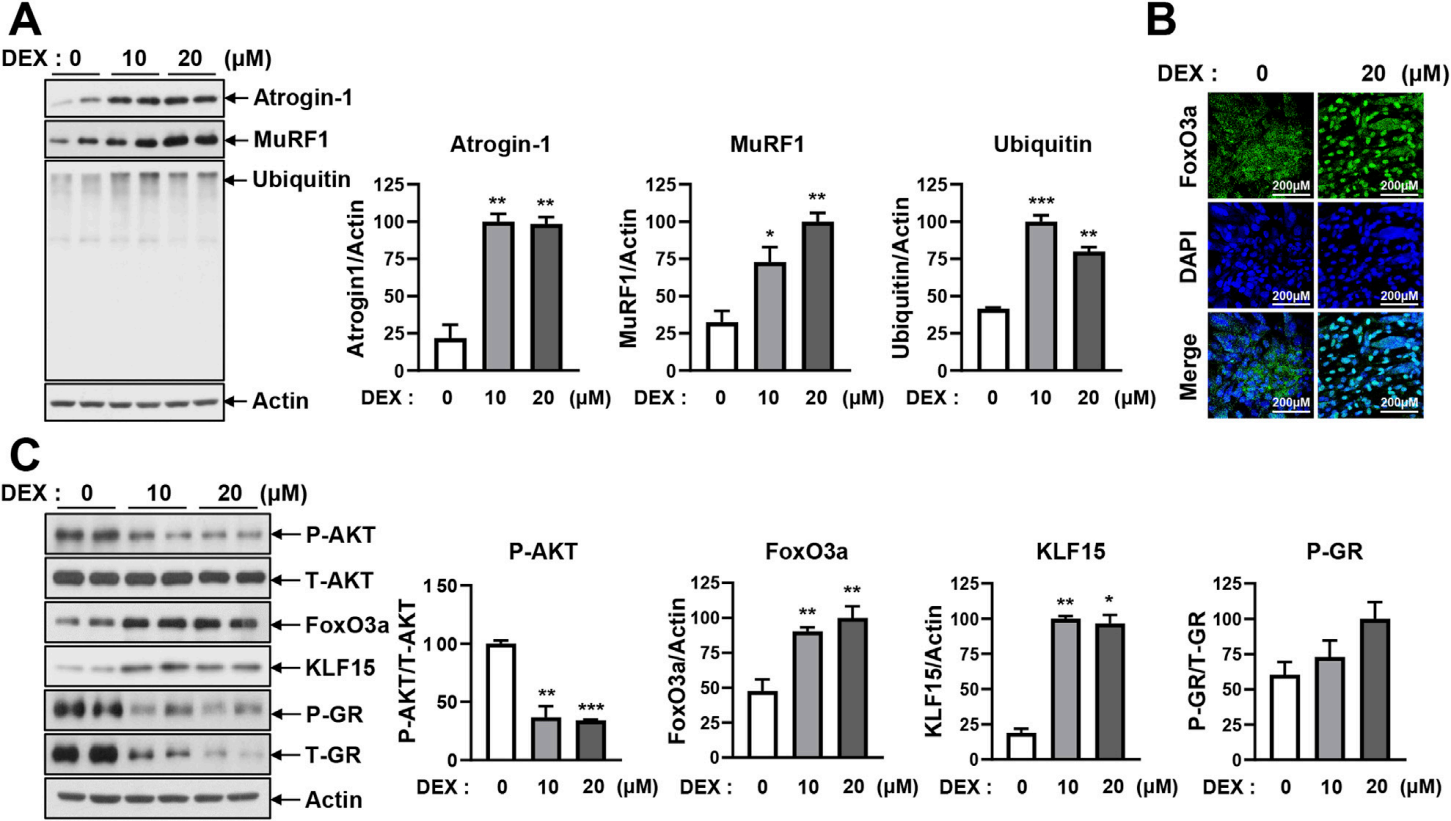
DEX induces atrophy in C2C12 myotubes by activating E3 ligase-mediated ubiquitination. **(A)** C2C12 myoblasts were differentiated for 5 days and then treated with 10 μM and 20 µM DEX for 24 h. Protein levels of atrogin-1, MuRF1, and ubiquitin were analyzed by immunoblotting. **(B)** C2C12 myoblasts were differentiated for 5 days and then treated with 20 µM DEX for 24 h. After treatment, the myotubes were stained with primary antibodies against FoxO3a, secondary antibodies, and DAPI. The nuclear translocation of FoxO3a was visualized by confocal microscopy. **(C)** C2C12 myoblasts were differentiated for 5 days and treated with 10 and 20 µM DEX for 24 h. Protein levels of p-AKT, FoxO3a, KLF15, and p-GR were analyzed by immunoblotting. Data are expressed as mean ± SEM. Statistical significance in **(A)** and **(C)** was determined using a t-test. *p < 0.05, **p < 0.01, ***p < 0.001 vs. CON.

Muscle atrophy is induced by several mechanisms, such as the inhibition of protein synthesis by endoplasmic reticulum (ER) stress, increased proteolytic activity caused by high Ca^2+^, induction of autophagy, activation of the caspase pathway, and stimulation of the UPS (Gallot and Bohnert, 2021; Hyatt et al., 2020). In the present study, these mechanisms were tested in differentiated DEX-treated C2C12 myotubes. To determine whether autophagy was involved in DEX-induced muscle atrophy, we tested the levels of sequestosome-1 (p62) and microtubule-associated protein 1 light chain 3 beta (LC3B) under the DEX treatment. No changes in p62 or LC3B were observed in response to DEX (Supplementary Figure 2A). No change in pro-caspase-3 or cleaved caspase-3 was detected in response to the DEX treatment (Supplementary Figure 2B). To determine whether protein synthesis inhibition by ER stress was involved in DEX-induced muscle atrophy, we analyzed phospho-eukaryotic initiation factor 2 (p-eIF2), activating transcription factor 4 (ATF4), phospho-calcium/calmodulin-dependent protein kinase kinase 2 (p-CaMKK2), and phospho-JNK (p-JNK). The levels of p-eIF2, ATF4, p-CaMKK2, and p-JNK did not change in response to the DEX treatment (Supplementary Figure 2C). Therefore, it was confirmed that muscle atrophy caused by DEX was not induced by autophagy, the caspase-3 pathway, or ER stress. Finally, the proteasome proteolysis pathway, which includes E3 ligase ubiquitinated proteins, was tested (Figure 2). The ubiquitin–proteasome pathway is a key mechanism of muscle atrophy, in which proteins are ubiquitinated by E1, E2, and E3 enzymes and subsequently degraded by the proteasome. Western blot analysis confirmed that DEX treatment increased the expression of atrogin-1, MuRF1, and ubiquitinated proteins in C2C12 myotubes (Figure 2A), indicating activation of the E3 ligase-mediated proteolytic system. Consistently, the mRNA levels of atrogin-1 and MuRF1 were also significantly increased in DEX-treated myotubes, as shown by quantitative reverse transcription polymerase chain reaction (qRT-PCR) analysis (Supplementary Figure 3A), supporting transcriptional upregulation of these E3 ligases.

To understand the upstream regulatory mechanism of atrogin-1 and MuRF1 expression, we examined the transcription factor FoxO3a, which is known to regulate these E3 ligases. FoxO3a translocates to the nucleus, where it activates its target genes. As shown in Figure 2B, FoxO3a was predominantly localized in the cytoplasm under control conditions but was clearly translocated to the nucleus following DEX treatment, as confirmed by DAPI counterstaining.

To investigate the regulatory pathway further, we analyzed the expression of phosphorylated AKT (p-AKT), FoxO3a, KLF15, and phosphorylated GR (p-GR), all of which are involved in GC signaling. Western blot analysis showed that DEX decreased p-AKT levels while increasing FoxO3a and KLF15 expression. By contrast, p-GR levels remained unchanged (Figure 2C; Supplementary Figure 3B).

Concomitantly, as shown in Figure 1, DEX treatment led to a significant elevation of intracellular iron levels in C2C12 myotubes. This increase coincided with reduced AKT phosphorylation and enhanced nuclear translocation of FOXO3a. These changes were associated with increased expression of atrogin-1 and MuRF1. Collectively, these findings suggest that DEX-induced muscle atrophy is mediated through activation of the intracellular iron–AKT–FOXO3a–E3 ligase signaling pathway, ultimately leading to enhanced protein degradation via the UPS.

### 3.3 DFO recovers DEX-induced myotube atrophy in differentiated C2C12 myotubes

**FIGURE 3:**
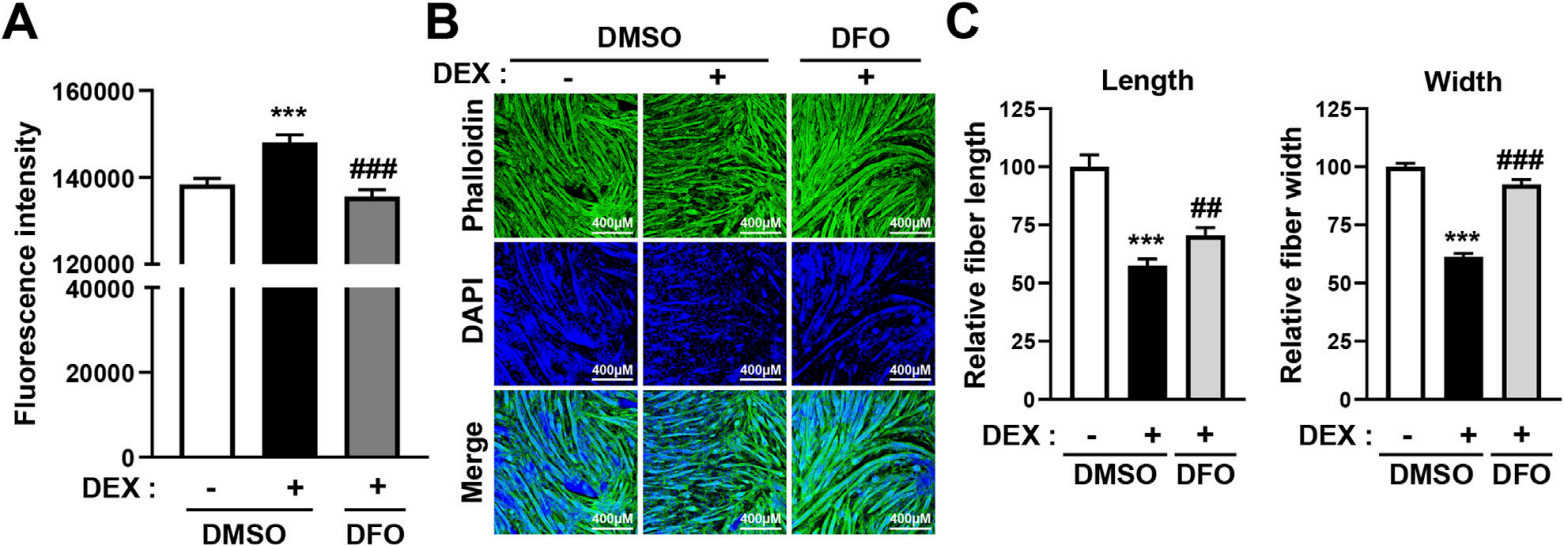
DFO treatment prevents DEX-induced atrophy of C2C12 myotubes. **(A)** C2C12 myoblasts were induced to differentiate for 5 days and then treated with 20 µM DEX with or without 400 µM DFO for 24 h. After treatment, intracellular iron levels were stained using fluorescent iron-staining dye and fluorescence intensity was quantified by spectrophotometry. **(B)** C2C12 myoblasts were induced to differentiate for 5 days and then treated with 20 µM DEX with or without 400 µM DFO for 24 h. Then, the myotubes were stained with phalloidin (actin fiber) and DAPI (nuclei). Myotube atrophy was observed by confocal microscopy, and **(B)** relative myotube length and width were measured by ImageJ software. Data are expressed as mean ± SEM. Statistical significance in **(A)** and **(B)** was determined using one-way ANOVA. ***p < 0.001 vs. CON; ^##^ *p* < 0.01, ^###^ *p* < 0.001 vs. DEX.

As shown in Figure 1, DEX treatment significantly increased intracellular iron levels in C2C12 myotubes. To determine whether reducing this iron accumulation could alleviate DEX-induced muscle atrophy, we co-treated differentiated C2C12 myotubes with DEX and DFO, an iron chelator, and measured the effects on intracellular iron levels. Fluorescence analysis revealed that DFO treatment effectively reduced the intracellular iron accumulation induced by DEX (Figure 3A; Supplementary Figure 4).

We then examined the effect of DFO on myotube morphology. As expected, DEX treatment resulted in a marked reduction in myotube length and width, indicative of atrophy. However, co-treatment with DFO significantly restored myotube size. Confocal microscopy revealed that DFO preserved the structural integrity of myotubes (Figure 3B), and quantitative analysis using ImageJ confirmed that both fiber length and width were significantly improved compared with DEX treatment alone (Figure 3C).

Importantly, DFO treatment alone did not alter myotube morphology under basal conditions, suggesting that DFO does not affect muscle homeostasis in the absence of DEX (Supplementary Figure 5). These findings indicate that DFO mitigates DEX-induced muscle atrophy, likely through the reduction of intracellular iron accumulation.

### 3.4 DFO treatment prevents DEX-induced atrogin-1 and MuRF1 expression by inhibiting FoxO3 and KLF15

**FIGURE 4:**
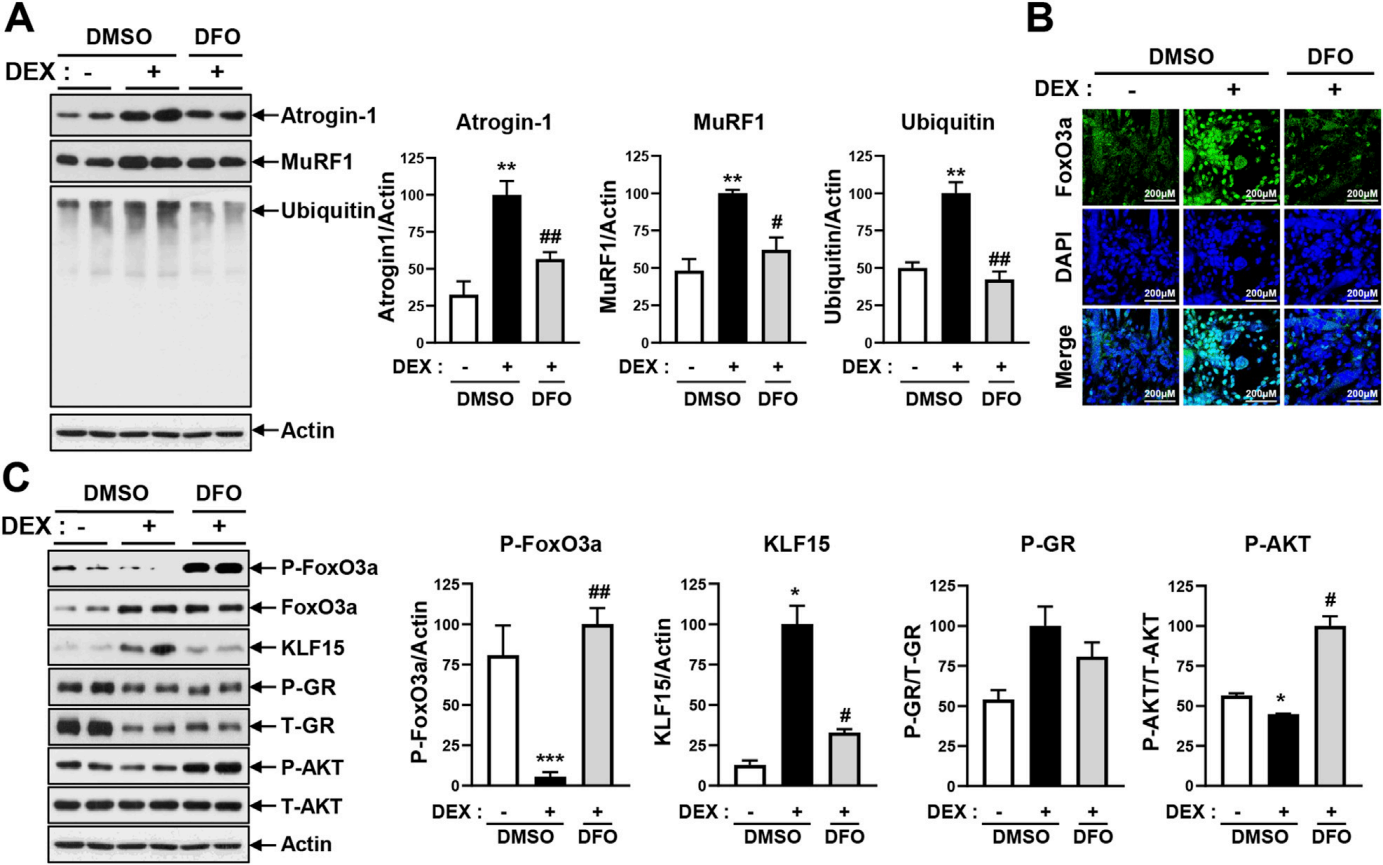
DFO treatment prevents DEX-induced atrogin-1 and MuRF1 expression. **(A)** Differentiated C2C12 myotubes were treated with 20 µM DEX with or without 400 µM DFO for 24 h. Levels of atrogin-1, MuRF1, and ubiquitin were analyzed by immunoblotting. **(B)** Differentiated C2C12 myotubes were treated with 20 µM DEX with or without 400 µM DFO for 48 h. Then, the myotubes were stained with FoxO3a. Nuclear translocation of Foxo3a was observed by confocal microscopy. **(C)** Differentiated C2C12 myotubes were treated with 20 µM DEX with or without 400 µM DFO for 48 h. Levels of p-FoxO3a, KLF15, p-GR, and p-AKT were analyzed by immunoblotting. Data are expressed as mean ± SEM. In **(A)** and **(C)**, significance was determined using one-way ANOVA. *p < 0.05, **p < 0.01, ***p < 0.001 vs. CON; ^#^ *p* < 0.05, ^##^ *p* < 0.01 vs. DEX.

DEX has been observed to induce myotube atrophy primarily through the E3 ligase–dependent proteasomal proteolysis pathway, as shown in Figure 2 and Supplementary Figure 2. In this pathway, the muscle-specific E3 ligases atrogin-1 and MuRF1 play a critical role in activating proteasomal protein degradation. This catabolic response was consistently accompanied by a marked increase in intracellular iron levels (Figures 1, 2). Given that DFO effectively reduced DEX-induced intracellular iron accumulation (Figure 3; Supplementary Figure 4), we subsequently investigated whether restoring iron homeostasis could suppress downstream proteolytic signaling. As shown in Figure 4A, co-treatment with DFO significantly reduced the expression of atrogin-1 and MuRF1, suggesting that iron chelation mitigates DEX-induced proteasomal degradation. The AKT/FOXO3a, GR/FOXO3a, and GR/KLF15 pathways are recognized transcriptional regulators of atrogin-1 and MuRF1. To investigate the impact of DFO on these signaling cascades, we analyzed FOXO3a activity. Immunofluorescence analysis revealed that DEX stimulated the nuclear translocation of FOXO3a, an effect that was significantly inhibited by DFO treatment (Figure 4B). Because FOXO3a activation is negatively regulated by AKT phosphorylation, we next assessed the phosphorylation status of both AKT and FOXO3a. DEX reduced the phosphorylation of both proteins, whereas DFO restored their phosphorylated levels, thereby preventing FOXO3a activation and its subsequent nuclear migration (Figure 4C).

In parallel, DEX-induced KLF15 expression was significantly decreased by DFO at both the protein (Figure 4C) and mRNA (Supplementary Figure 6A) levels, while GR levels remained unchanged. Because KLF15 also functions as a transcriptional activator of atrogin-1 and MuRF1, we further assessed the mRNA expression of these E3 ligases. DFO significantly reduced their transcript levels, consistent with its observed inhibitory effects on both FOXO3a and KLF15 (Supplementary Figure 6B).

To assess further whether DFO-mediated restoration of AKT activity influences downstream anabolic signaling, we evaluated components of the mTORC1 pathway, including phosphorylated S6 kinase (p-S6K) and phosphorylated 4E-BP1 (p-4E-BP1). As shown in Supplementary Figure 7, p-S6K levels remained unchanged between the control and DEX-treated groups but were notably increased following DFO co-treatment. In contrast, p-4E-BP1 levels were reduced by DEX and subsequently restored upon DFO treatment.

Collectively, these findings indicate that DFO inhibits DEX-induced myotube atrophy by restoring the intracellular iron balance, preserving AKT activity, and suppressing both FOXO3a nuclear translocation and KLF15 expression. These effects ultimately lead to reduced activation of E3 ligases and attenuation of proteasome-mediated protein degradation.

### 3.5 DFO alleviates DEX-induced muscle atrophy in C57BL/6J mice

**FIGURE 5:**
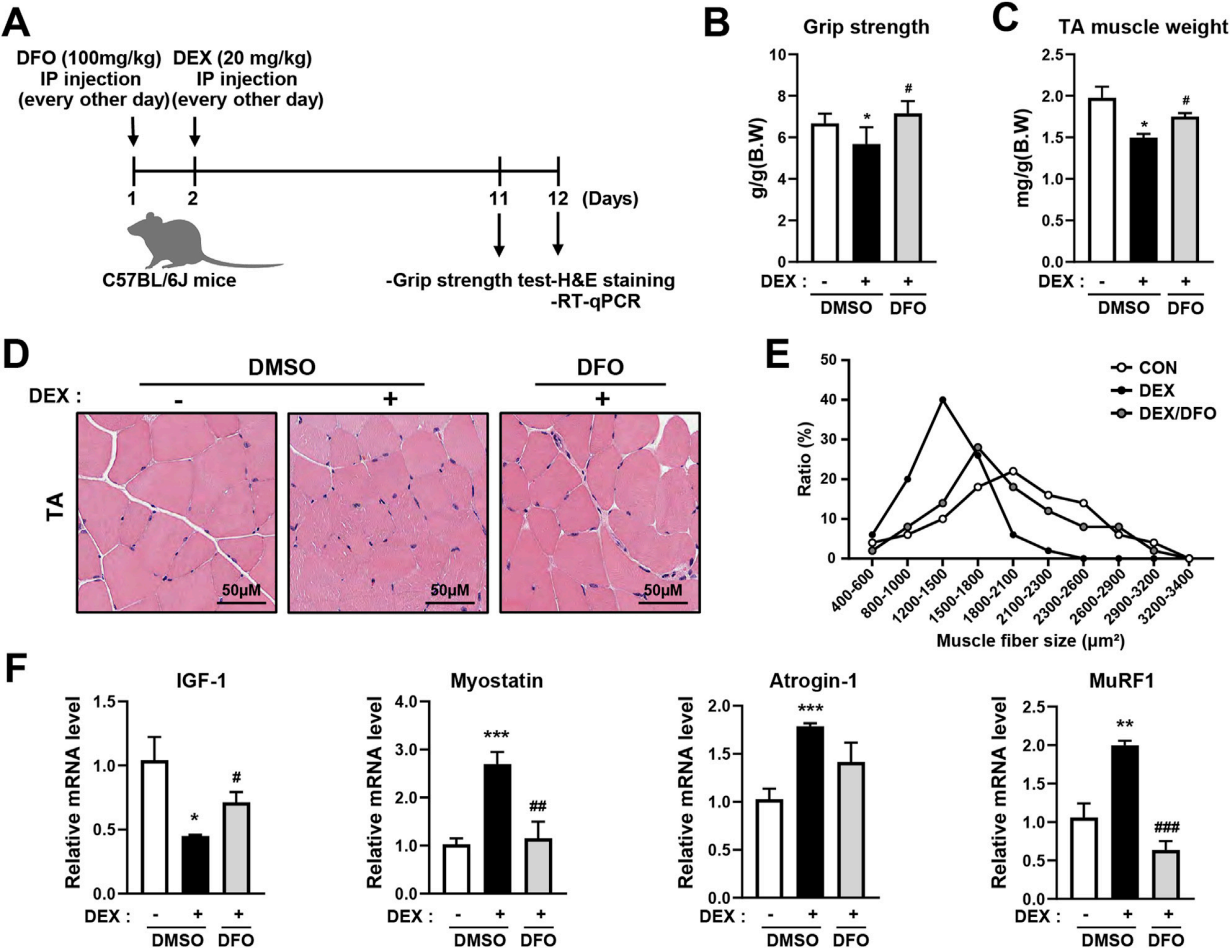
DFO prevents DEX-induced muscle atrophy in C57BL/6J mice. **(A)** Male C57BL/6J mice were injected with 100 mg/kg DFO and 20 mg/kg DEX every other day for 10 days. **(B)** Grip strength tests were performed on day 10, and the mice were sacrificed on day 11 to isolate the TA muscle. **(C)** Isolated TA muscle weight was measured. **(D,E)** The cross-sectional area of the TA muscle was analyzed using ImageJ software after H&E staining. **(F)** Muscle synthesis and degradation-related gene expression were analyzed by qRT-PCR. Data are expressed as mean ± SEM. Statistical significance in **(B)**, **(C)**, and **(F)** was determined using one-way ANOVA. *p < 0.05, **p < 0.01, ***p < 0.001 vs. CON; ^#^ *p* < 0.05, ^##^ *p* < 0.01, ^###^ *p* < 0.001 vs. DEX.

To improve the effects of DFO on DEX-induced muscle atrophy in C57BL/6J mice, C57BL/6J mice were divided into a control group, a DEX-only treatment group, and a DEX + DFO combined treatment group. Mice were treated with DEX with or without DFO by intraperitoneal injection every other day for 10 days (Figure 5A). Grip strength was measured on day 10 (Figure 5B). Tibialis anterior (TA) muscle mass of the hind limbs was measured on day 11 (Figure 5C). DFO-treated mice recovered grip strength and TA muscle weight.

GCs induce fast-twitch or muscle atrophy in type 2 muscle fibers, with little or no effect on type 1 muscle fibers. Muscle atrophy caused by GC is more effectively evoked in the TA, which is a fast-twitch glycolytic muscle, whereas the soleus muscle is an oxidative muscle. In this study, to improve DFO-recovered TA atrophy in DEX-treated mice, the cross-sectional size of the TA muscle was determined using hematoxylin and eosin (H&E) staining (Figure 5D). The DEX-only treatment group had a smaller TA muscle cross-sectional size than the control group, and the DEX + DFO combined treatment group had an improved TA muscle cross-sectional size versus the DEX-only treatment group (Figure 5E).

In the DEX muscle atrophy model, DFO improved body weight, grip strength, TA muscle weight, and TA muscle cross-sectional size. It has been reported that muscle loss is controlled by various factors, such as a decrease in insulin-like growth factor-1 (IGF-1), a protein synthesis-inducing factor, and an increase in myostatin, a protein degradation-inducing factor. To determine whether DFO regulates IGF-1 or myostatin, the RNA expression levels of IGF-1 and myostatin were measured by qRT-PCR. DEX treatment significantly decreased IGF-1 and increased myostatin expression, whereas DFO administration effectively reversed these changes (Figure 5F). Furthermore, the DEX-induced upregulation of the muscle-specific E3 ubiquitin ligases atrogin-1 and MuRF1 were attenuated by DFO treatment (Figure 5F). As a result, the DFO treatment alleviated DEX-induced muscle atrophy, reflected in grip strength, TA muscle weight, and TA muscle cross-sectional size, by reducing atrogin-1 and MuRF1 expression.

## 4 Discussion

GCs are extensively used for their potent anti-inflammatory and immunosuppressive properties. However, their prolonged use often leads to skeletal muscle atrophy, a debilitating side effect with limited therapeutic options. While the activation of UPS is a well-established mechanism underlying GC-induced muscle atrophy, the contribution of altered iron homeostasis remains poorly understood. In this study, we investigated whether DEX-induced muscle atrophy is associated with disruptions in iron homeostasis and whether treatment with the DFO, an iron chelator, could alleviate these effects in both differentiated myotubes and a murine model.

Muscle atrophy is a multifactorial process driven by excessive protein degradation and reduced synthesis. In our model, DEX markedly upregulated the expression of atrogin-1 and MuRF1, two E3 ubiquitin ligases involved in proteasomal degradation, consistent with previous studies of GC-induced catabolism (Bodine et al., 2001; Sandri et al., 2004). FOXO3a, a key transcription factor that regulates these ligases, was activated by DEX, as evidenced by its nuclear translocation. These findings align with reports showing that FOXO3a activation promotes skeletal muscle breakdown (Milan et al., 2015). Although autophagy can also contribute to muscle atrophy under certain stress conditions, such as nutrient deprivation or proteotoxic stress (Cohen-Kaplan et al., 2016; Lim et al., 2015), our DEX model did not significantly alter autophagy-related protein levels, suggesting that the UPS remained the dominant catabolic pathway in this context.

A key novel insight from this study is the identification of intracellular iron overload as a causative driver of DEX-induced muscle atrophy. We observed that DEX elevated intracellular iron levels, and DFO co-treatment effectively reversed this increase. These results are consistent with our previous findings (Cui et al., 2019), demonstrating that iron loading with FeSO_4_ or FeCl_3_ can directly induce muscle atrophy. Similarly, Ikeda et al. (2016) showed that iron accumulation suppresses Akt phosphorylation, leading to enhanced FOXO3a activity and activation of E3 ligases. Taken together, these data suggest that iron overload is not merely a secondary consequence of DEX exposure but serves as a primary upstream factor initiating proteolytic signaling.

Importantly, we found that DFO restored Akt phosphorylation while reducing FOXO3a nuclear localization and E3 ligase expression. This suggests that DFO exerts its protective effect, at least in part, through reactivation of the Akt–FOXO3a axis—a pathway known to balance muscle protein turnover. While DEX appears to upregulate FOXO3a via GR-mediated transcriptional activation, iron overload likely contributes to impaired Akt signaling, further enhancing FOXO3a activity. The precise mechanism by which DFO restores Akt phosphorylation remains to be elucidated. It is possible that DFO-mediated iron chelation reduces oxidative or inflammatory stress, such as p-JNK activation, thereby enabling reactivation of the PI3K/Akt pathway. However, further studies are required to understand fully how iron chelation influences upstream regulators of Akt in the context of GC-induced muscle atrophy.

The therapeutic efficacy of DFO was also confirmed *in vivo*, as DFO-treated mice exhibited improved grip strength, preserved TA muscle mass, and increased muscle fiber cross-sectional area. Furthermore, DFO partially restored the balance between anabolic and catabolic regulators by increasing IGF-1 and reducing myostatin expression, two key modulators of muscle mass homeostasis. These findings are consistent with Bose et al. (2025), who showed that iron chelation protects against sarcopenia in Klotho-deficient mice by improving mitochondrial function and reducing oxidative stress. However, as iron is essential for mitochondrial respiration and DNA synthesis, overchelation may impair myogenic function. Indeed, other studies suggest that iron deficiency can also cause muscle atrophy by reducing myoblast proliferation and mitochondrial activity (Vinke et al., 2023; Che et al., 2023). These observations emphasize the need for carefully titrated iron modulation strategies in therapeutic applications.

From a structural perspective, the distinct mechanisms of DEX and DFO can be better understood through their structure–activity relationships. DEX, a synthetic GC, possesses a specific steroid structure that allows it to bind to the GR with high affinity. This GR binding initiates a cascade of events leading to the upregulation of catabolic pathways, including the UPS, and ultimately contributing to muscle atrophy. By contrast, DFO possesses a structure characterized by its hexadentate siderophore motif, giving it high affinity for iron ions and allowing it to effectively chelate and remove excess iron from the cellular environment (Richardson, 2002). This iron-chelating property is directly related to its protective effects against DEX-induced atrophy, as DFO effectively counteracts the intracellular iron overload triggered by DEX treatment, thereby preventing downstream proteolytic signaling. These structural features are closely linked to the functional effects observed in this study—DFO’s chelation of excess iron directly mitigates the atrophic processes initiated by DEX.

In addition to its direct effects on myofibers, skeletal muscle regeneration also depends on satellite cell function. Previous studies have shown that both angiotensin II and glucocorticoids such as DEX inhibit satellite cell activation, proliferation, and differentiation, thereby impairing muscle regeneration (Rezk et al., 2012; Yoshida et al., 2013; Syverud et al., 2016; Dong et al., 2013). Moreover, DEX has also been shown to interact with the renin–angiotensin system, altering Ang II levels and receptor expression in various tissues (Zhan et al., 2024; Chansel et al., 1996; Ullian et al., 1996), which may further potentiate the inhibitory effects of Ang II on satellite cells. Although the direct impact of DFO on satellite cell dynamics remains poorly characterized, particularly in the context of concurrent glucocorticoid and Ang II exposure, its ability to restore intracellular iron homeostasis may offer indirect protective effects. Given that iron dysregulation can influence regenerative capacity, it is possible that DFO mitigates the detrimental consequences of DEX and Ang II on satellite cells by alleviating iron-mediated stress. Further studies are needed to elucidate the role of iron chelation in preserving satellite cell function during GC-induced muscle atrophy.

## 5 Conclusion

This study demonstrates that DEX-induced muscle atrophy is mediated by both activation of the UPS and disruption of intracellular iron homeostasis. Treatment with DFO effectively mitigated these effects by restoring iron balance and reactivating the Akt–FOXO3a signaling axis. These findings highlight the central role of iron dysregulation in GC-induced muscle atrophy and support the potential of iron chelation as a therapeutic strategy.

## Data Availability

The original contributions presented in the study are included in the article/Supplementary Material, further inquiries can be directed to the corresponding author.
