# Supplementary figures and images for: Deferoxamine prevents dexamethasone-induced muscle atrophy by reducing MuRF1 and atrogin-1
Source: Front Pharmacol. 2025 Jul 9;16:1582216. doi: 10.3389/fphar.2025.1582216 (PMC12283682; doi:10.3389/fphar.2025.1582216)

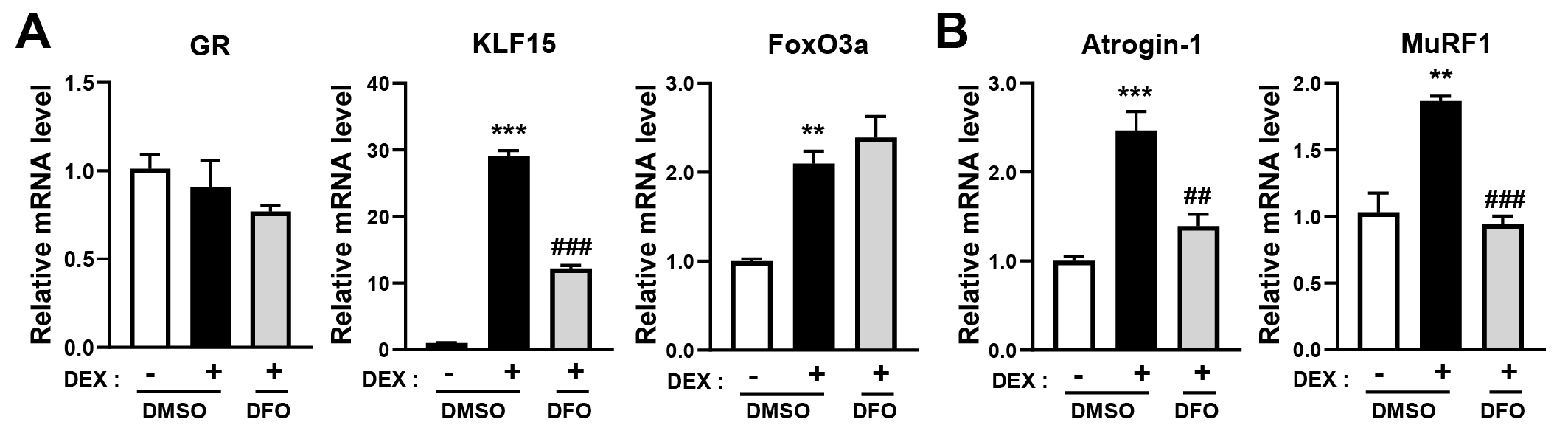

Supplement: Supplementary file 1 [file Image6.tif]

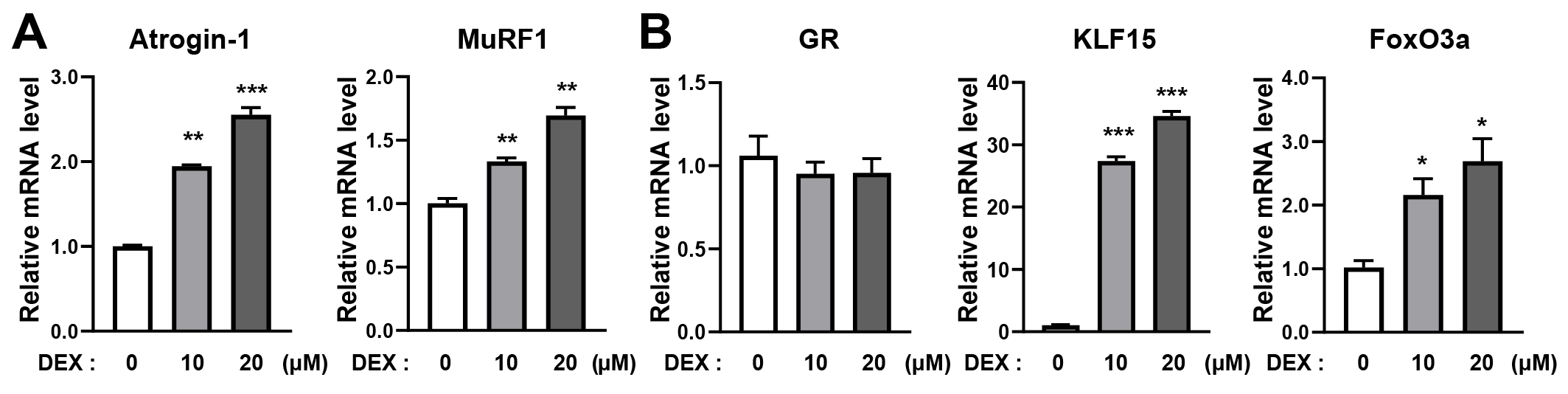

Supplement: Supplementary file 3 [file Image3.tif]

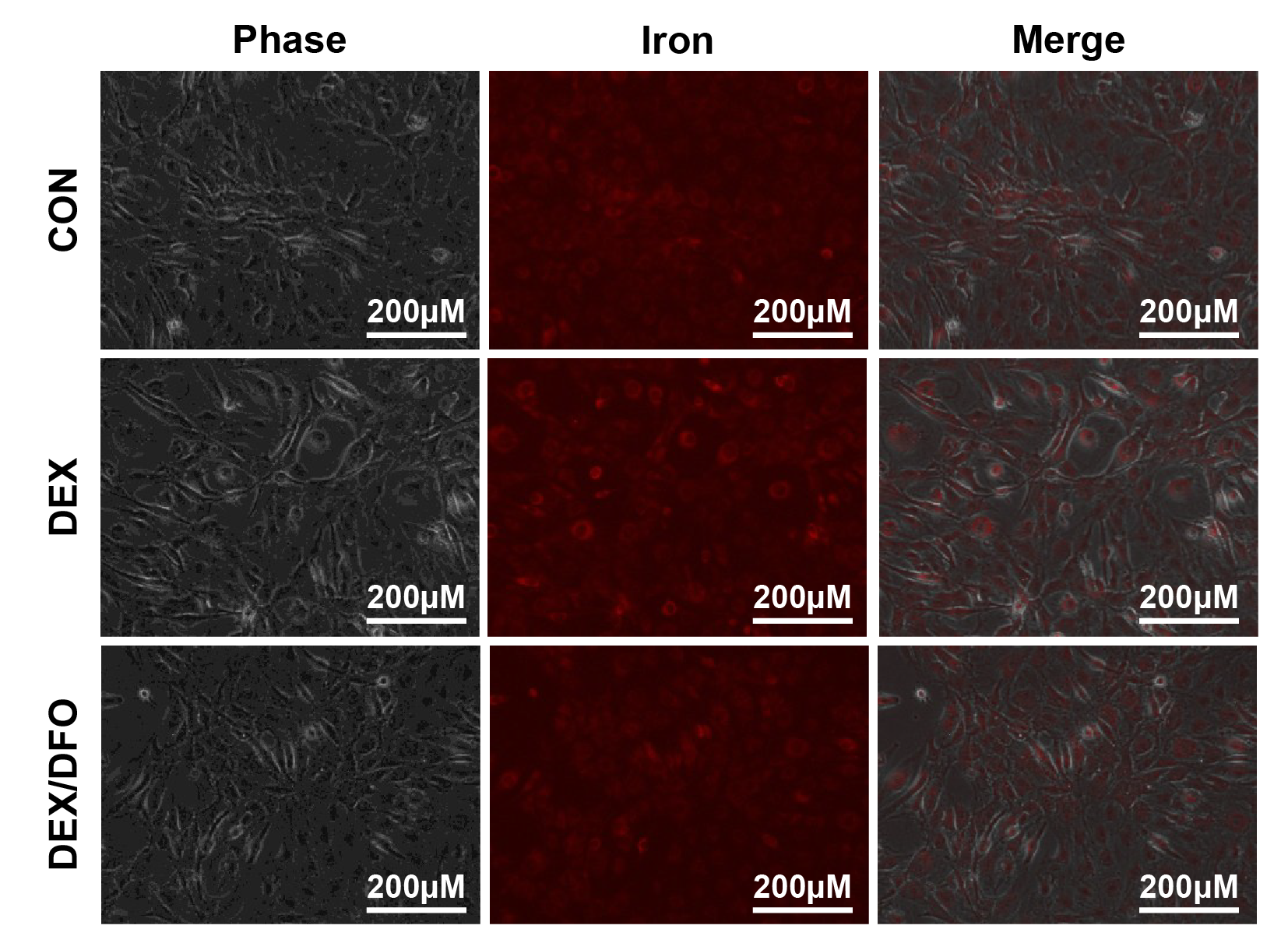

Supplement: Supplementary file 4 [file Image4.tif]

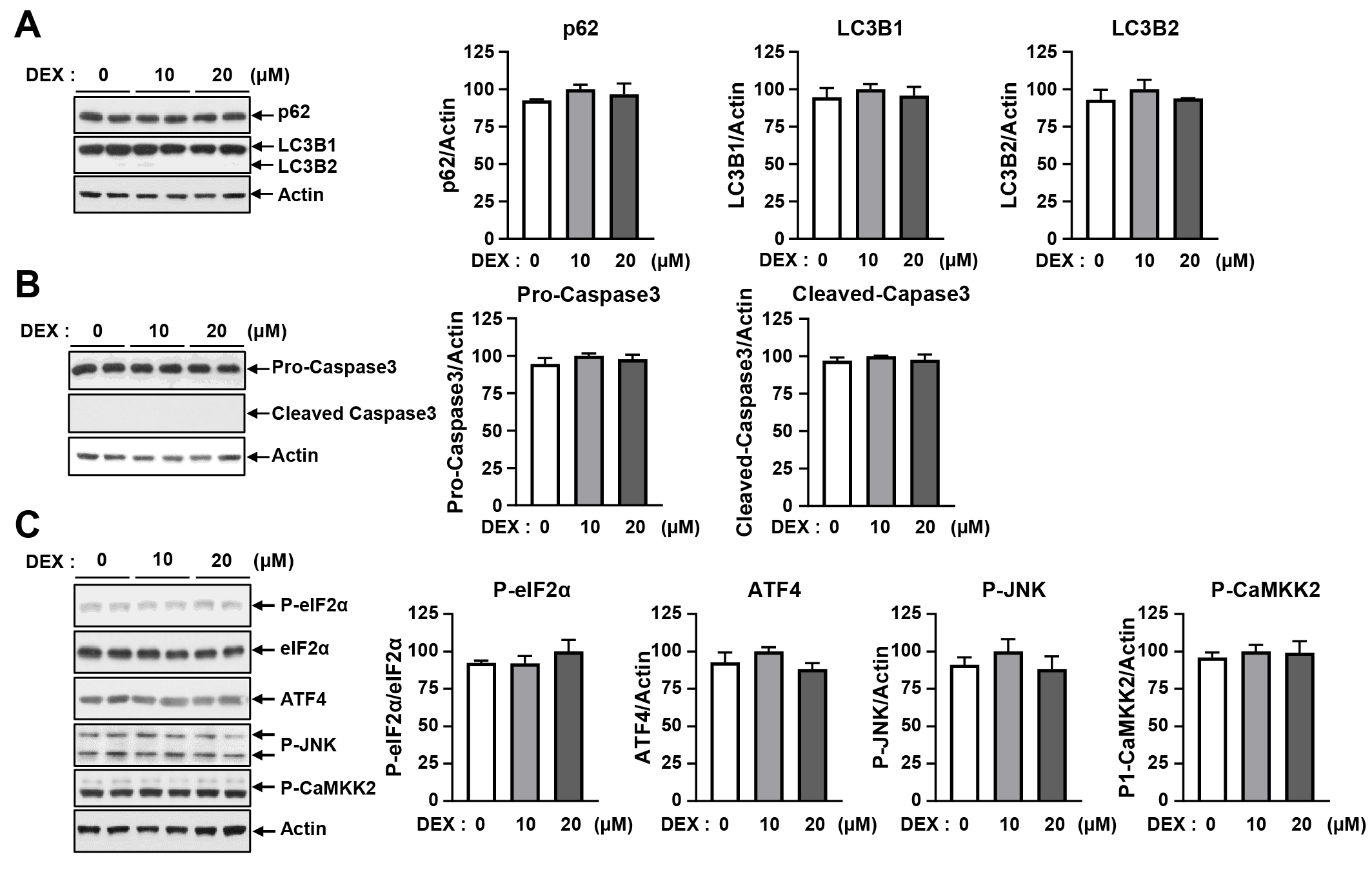

Supplement: Supplementary file 5 [file Image2.tif]

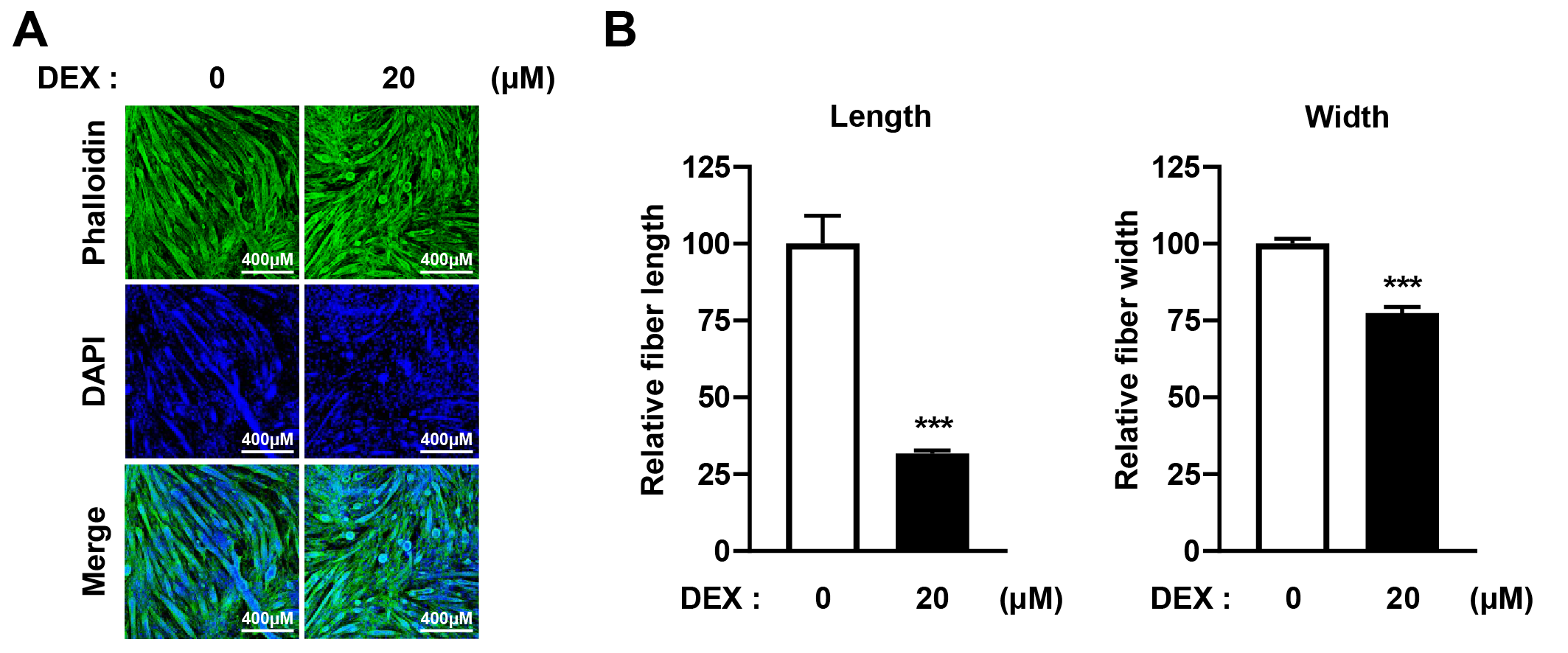

Supplement: Supplementary file 6 [file Image1.tif]

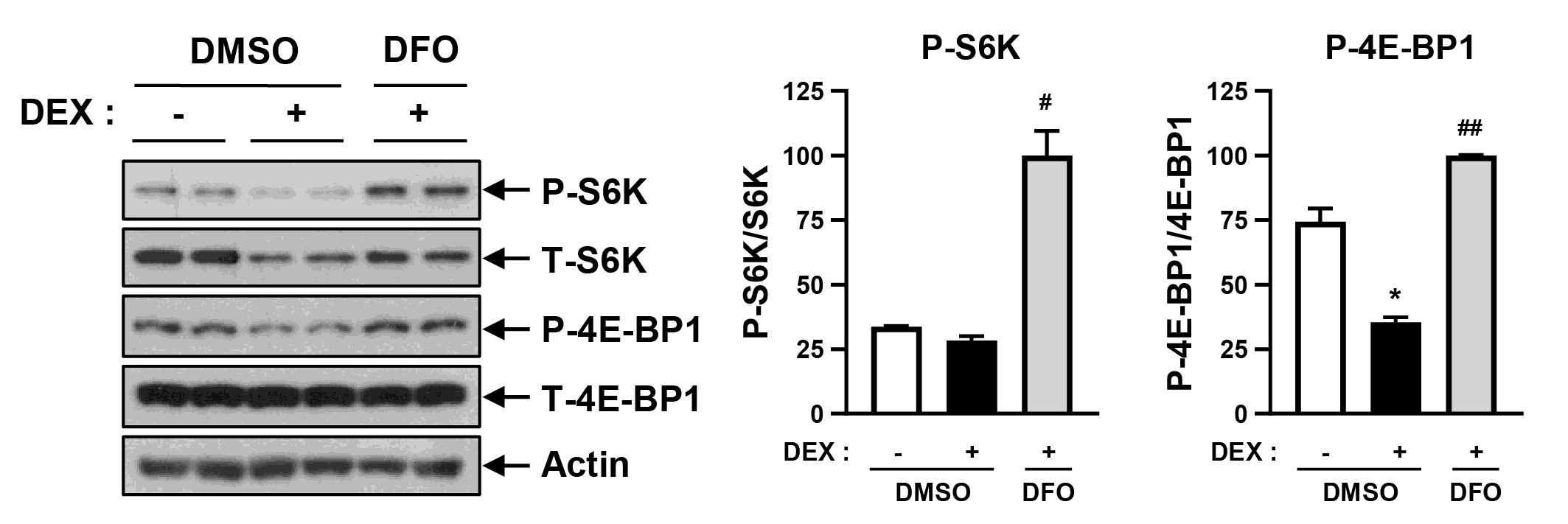

Supplement: Supplementary file 7 [file Image7.tif]

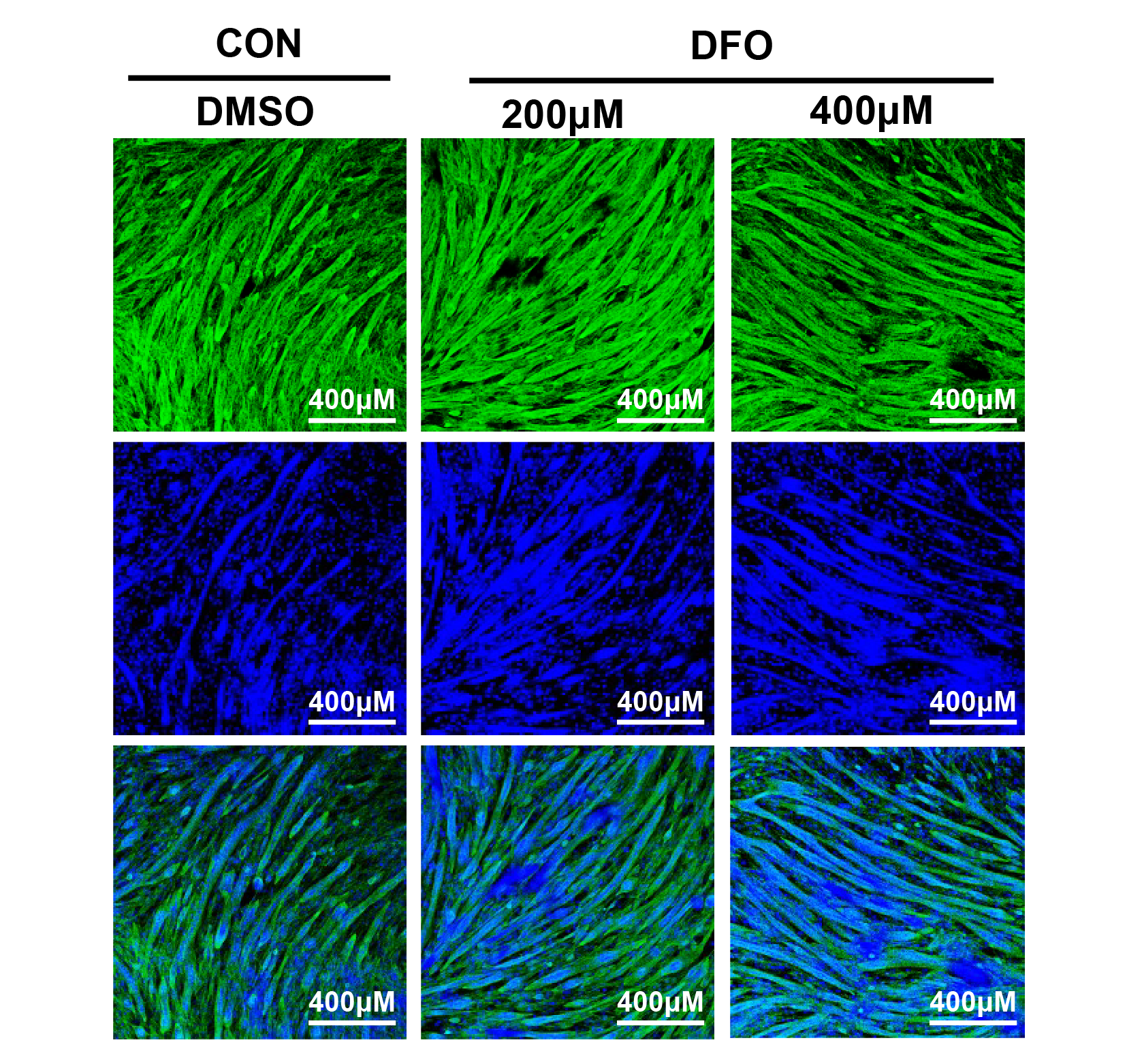

Supplement: Supplementary file 8 [file Image5.tif]
