# Supplementary material for: Deferoxamine prevents dexamethasone-induced muscle atrophy by reducing MuRF1 and atrogin-1
Source: Front Pharmacol. 2025 Jul 9;16:1582216. doi: 10.3389/fphar.2025.1582216 (PMC12283682; doi:10.3389/fphar.2025.1582216)
Supplement: Supplementary file 2 [file Supplementaryfile1.docx]

Supplementary Material

# Supplementary Figures and Tables

## Supplementary Figure. 1

**
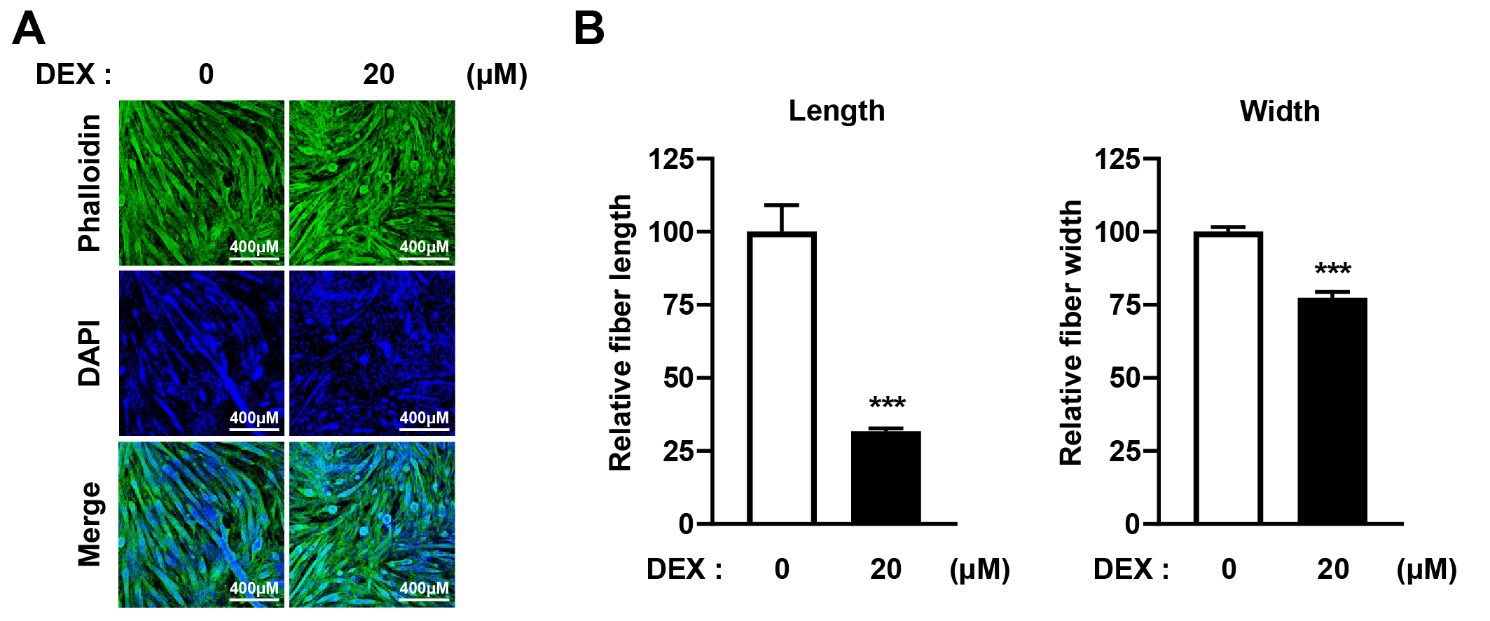
**

**Supplementary Figure. 1 C2C12 myotube atrophy is evoked by DEX treatment.** (A) C2C12 myoblasts were induced to differentiate for 5 days and then treated with 20 µM DEX for 24 hours. After treatment, the myotubes were stained with phalloidin (actin fiber) and DAPI (nuclei). Myotube atrophy was observed by confocal microscopy, and (B) relative myotube length and width were measured by ImageJ software. Data are expressed as mean ± SEM. Statistical significance in (B) was determined using a t-test. ***p < 0.001 vs. control (CON).

## Supplementary Figure. 2


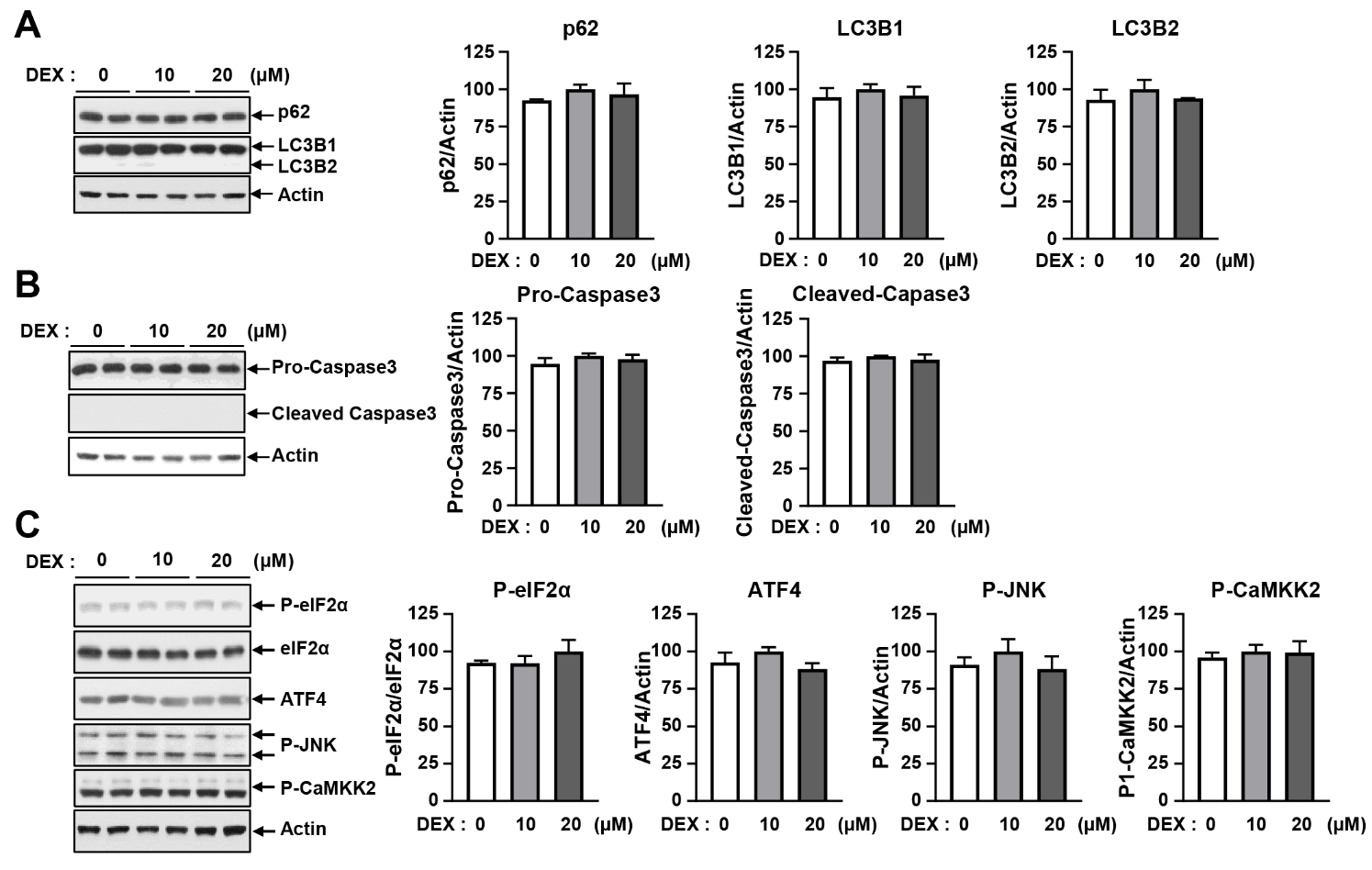


**Supplementary Figure. 2 DEX does not induce muscle atrophy through autophagy, cell death, or calcium ion-mediated ER stress.** (A) C2C12 myoblasts were differentiated for 5 days and treated with 10 µM and 20 µM DEX for 24 hours. Autophagy-related protein levels were analyzed by immunoblotting. (B) Cell death-related protein levels were analyzed by immunoblotting. (C) ER stress-related protein levels were analyzed by immunoblotting. Data are expressed as mean ± SEM.

## Supplementary Figure. 3


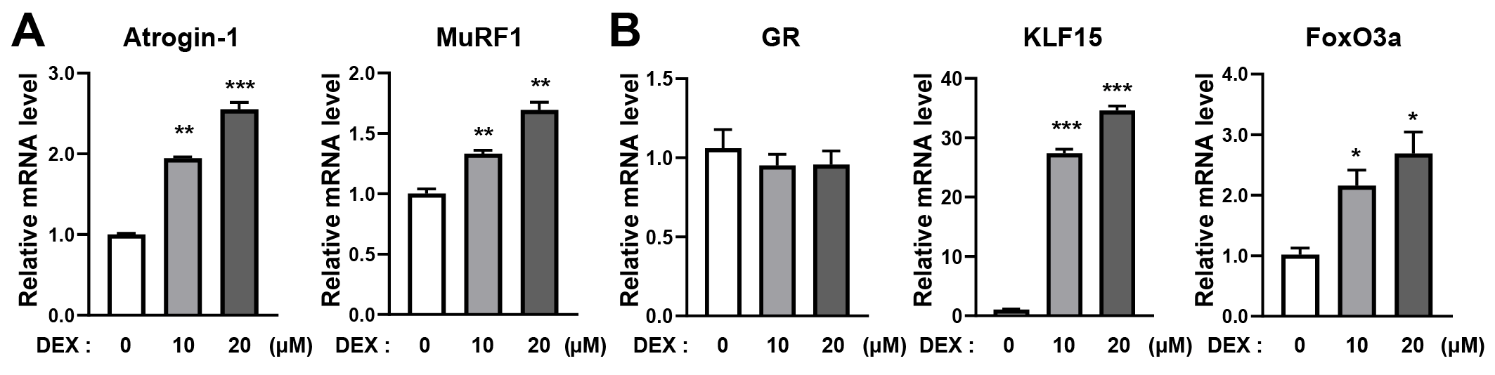


**Supplementary Figure. 3 DEX induces E3 ligase gene expression in C2C12 myotubes.** C2C12 myoblasts were induced to differentiate for 5 days and then treated with 10 µM and 20 µM DEX for 24 hours. Expression of (A) E3 ligase genes and (B) transcription factor genes was analyzed by qRT-PCR. Data are expressed as mean ± SEM. Statistical significance in (A) and (B) was determined using a t-test. *p < 0.05, **p < 0.01, ***p < 0.001 vs. CON.

## Supplementary Figure. 4


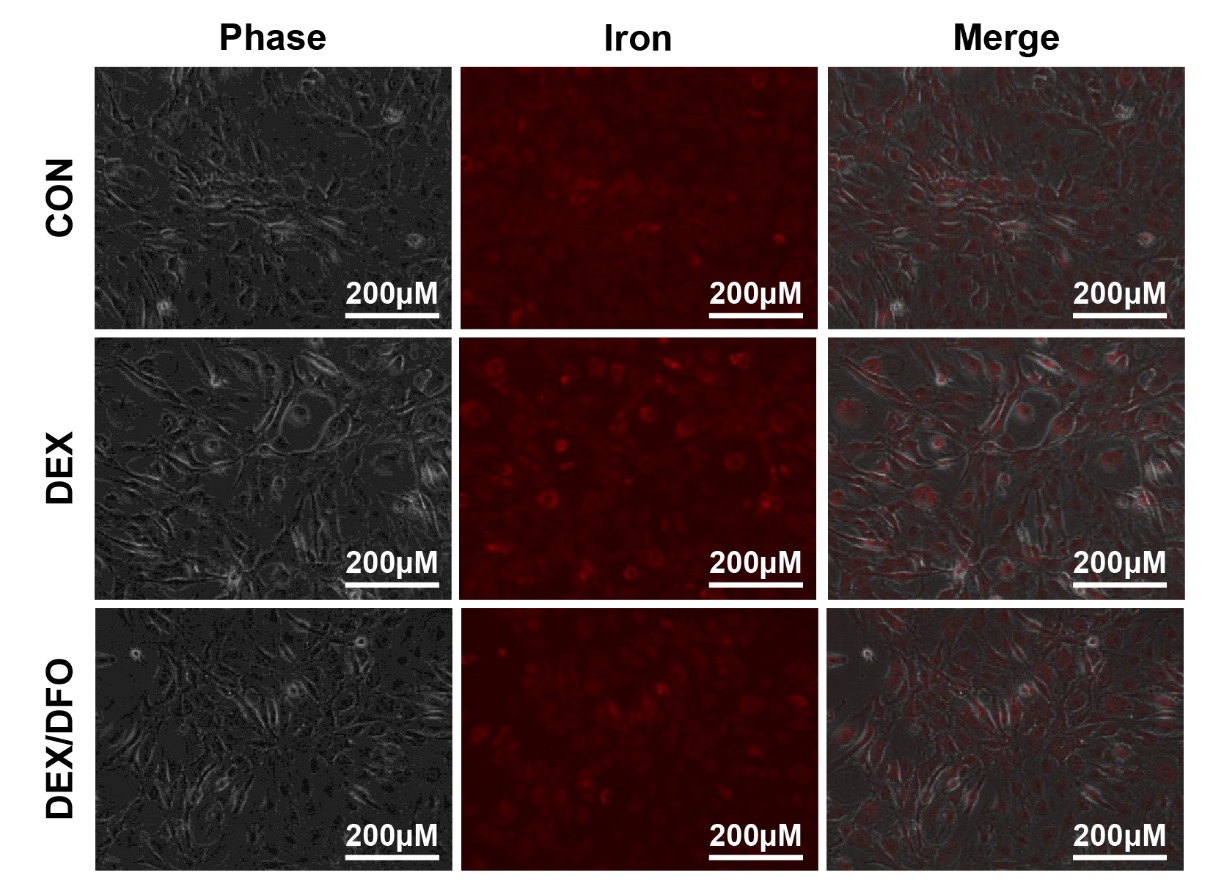


**Supplementary Figure. 4 DFO treatment reduces DEX-induced intracellular iron accumulation of C2C12 myotubes.** C2C12 myoblasts were induced to differentiate for 5 days and then treated with 200 µM and 400 µM DFO for 24 hours. After treatment, intracellular iron levels were visualized using a fluorescent iron-staining dye and fluorescence microscopy.

## Supplementary Figure. 5


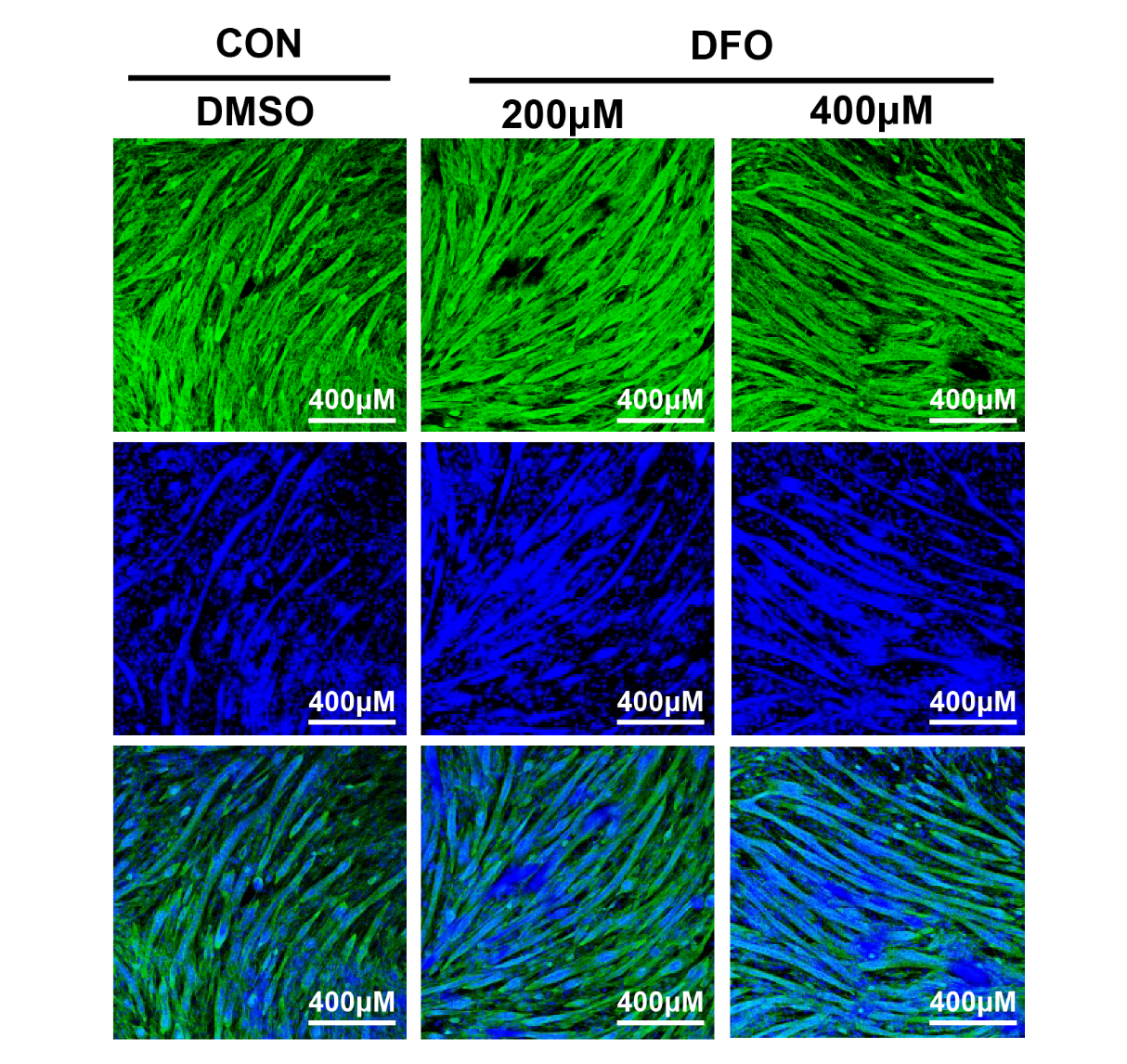


**Supplementary Figure. 5 DFO alone does not alter C2C12 myotubes morphology.** C2C12 myoblasts were induced to differentiate for 5 days and then treated with 200 µM and 400 µM DFO for 24 hours. Then, the myotubes were stained with phalloidin (actin fiber) and DAPI (nuclei). The myotubes were observed by confocal microscopy.

## Supplementary Figure. 6


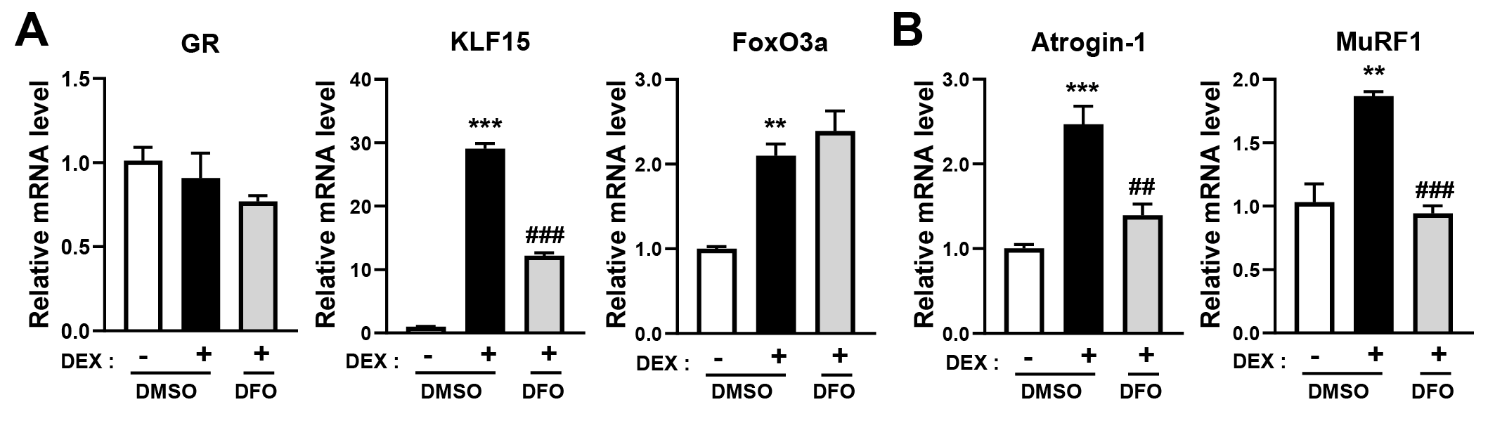


**Supplementary Figure. 6 DFO inhibits E3 ligase gene expression and the E3 ligase regulator in DEX-treated C2C12 myotubes.** Differentiated C2C12 myotubes were treated with 20 µM DEX with or without 400 µM DFO for 24 hours. Expression of (A) transcription factor gene and (B) E3 ligase gene was analyzed by qRT-PCR. Data are expressed as mean ± SEM. Significance was determined using ono-way ANOVA. **p < 0.01, ***p < 0.001 vs. CON; **^##^**p < 0.01, **^###^**p < 0.001 vs. DEX.

## Supplementary Figure. 7


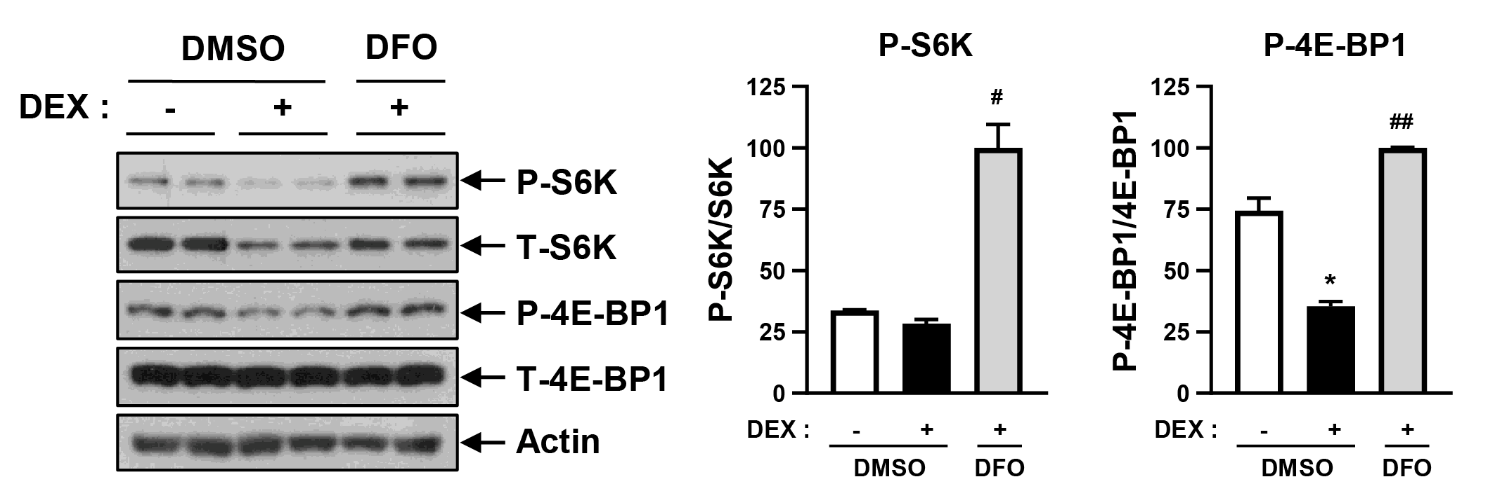


**Supplementary Figure. 7 DFO restores 4E-BP1 phosphorylation and enhances S6K phosphorylation in C2C12 myotubes.** Differentiated C2C12 myotubes were treated with 20 µM DEX with or without 400 µM DFO for 24 hours. Protein levels of p-S6K and p-4E-BP1 were analyzed by immunoblotting. Data are expressed as mean ± SEM. Significance was determined using ono-way ANOVA. *p < 0.05 vs. CON; **^#^**p < 0.05, **^##^**p < 0.01 vs. DEX.

## Supplementary Table. 1

**Sequences of PCR primers**

| **Name** | **Sequence (5’-)** | **Supplier** |
| --- | --- | --- |
| Mouse GR  (Forward primer) | **ACAGACTTTCGGCTTCTGGA** | Bioneer, Korea |
| Mouse GR  (Reverse primer) | **CTTCTCTGTCGGGGTAGCAC** | Bioneer, Korea |
| Mouse FoxO3a  (Forward primer) | **CTTCCCATATACCGCCAAGA** | Bioneer, Korea |
| Mouse FoxO3a  (Reverse primer) | **TGGATAGTCTGCATGGGTGA** | Bioneer, Korea |
| Mouse Atrogin1  (Forward primer) | **GCAAACACTGCCACATTCTCTC** | Bioneer, Korea |
| Mouse Atrogin1  (Reverse primer) | **CTTGAGGGGAAAGTGAGACG** | Bioneer, Korea |
| Mouse MuRF1  (Forward primer) | **AGGACTGGTGCAGAGTGACCAA** | Bioneer, Korea |
| Mouse MuRF1  (Reverse primer) | **TTCTCGTCCAGGATGGCGTA** | Bioneer, Korea |
| Mouse IGF-1  (Forward primer) | **TGGATGCTCTTCAGTTCGTG** | Bioneer, Korea |
| Mouse IGF-1  (Reverse primer) | **GCAACACTCATCCACAATGC** | Bioneer, Korea |
| Mouse KLF15  (Forward primer) | **CCAAACCTATTGGCTCAGGA** | Bioneer, Korea |
| Mouse KLF15  (Reverse primer) | **GCAACACTCATCCACAATGC** | Bioneer, Korea |
| Mouse Myostatin  (Forward primer) | **CTGTAACCTTCCCAGGACCA** | Bioneer, Korea |
| Mouse Myostatin  (Reverse primer) | **GCAGTCAAGCCCAAAGTCTC** | Bioneer, Korea |
| Mouse 36B4  (Forward primer) | **CACCGAGGCAACAGTTGG** | Bioneer, Korea |
| Mouse 36B4  (Reverse primer) | **CGACTCACAGAGCAGGC** | Bioneer, Korea |
